# The NBA and Youth Basketball: Recommendations for Promoting a Healthy and Positive Experience

**DOI:** 10.1007/s40279-018-0950-0

**Published:** 2018-06-30

**Authors:** John P. DiFiori, Arne Güllich, Joel S. Brenner, Jean Côté, Brian Hainline, Edward Ryan, Robert M. Malina

**Affiliations:** 1 Primary Care Sports Medicine, Hospital for Special Surgery, 535 East 70th Street, New York, NY 10021 USA; 2 Department of Sports Science, University of Kaiserslautern, Kaiserslautern, Germany; 3 Department of Pediatrics, Children’s Hospital of The King’s Daughters Eastern Virginia Medical School, Norfolk, VA USA; 4 School of Kinesiology and Health Studies, Queens University, Kingston, ON Canada; 5 NCAA, Indianapolis, IN USA; 6 USA Basketball, Colorado Springs, CO USA; 7 Department of Kinesiology and Health Education, University of Texas, Austin, TX USA

## Abstract

Participation in sports offers both short-term and long-term physical and psychosocial benefits for children and adolescents. However, an overemphasis on competitive success in youth sports may limit the benefits of participation, and could increase the risk of injury, burnout, and disengagement from physical activity. The National Basketball Association and USA Basketball recently assembled a group of leading experts to share their applied research and practices to address these issues. This review includes the group’s analysis of the existing body of research regarding youth sports participation and the related health, performance, and psychosocial outcomes. Based upon this, age-specific recommendations for basketball participation are provided that aim to promote a healthy and positive experience for youth basketball players.

## Key Points

**Table:** 

Participation in sports offers both short-term and long-term physical and psychosocial benefits for children and adolescents.
Youth should be provided opportunities and encouraged to sample different sports. They should avoid specializing in basketball prior to age 14 years.
The NBA and USA Basketball have developed age-specific guidelines for basketball participation for young athletes that are intended to promote a healthy and positive youth basketball experience.

## Background

Participation in youth sports such as basketball offers many potential benefits for children and adolescents. Youth sport participation provides an avenue to develop peer relationships, self-esteem, and leadership qualities [1]. It may also lay the foundation for an active and healthier adult lifestyle [2–4]. Basketball has one of the highest rates of youth sport participation and is well suited to offer young athletes opportunities to obtain these benefits.

However, an overemphasis on competitive success in youth sports may impede children from realizing the benefits of participation, and may ultimately limit their ability to reach their athletic potential. Such a highly-competitive approach may be driven by desires for children to gain placement on elite travel teams, secure high school roster spots, obtain collegiate scholarships, and eventually earn professional contracts. This focus on early results rather than playing sport for enjoyment and the long-term physical and psycho-social benefits has led to several well-recognized issues:

1. Pressure to begin high-intensity training in childhood.
2. Single-sport specialization that occurs prior to adolescence.
3. Frequent and multiple competitive event scheduling.
4. Increased risk for injury, burnout, and disengagement from health-promoting physical activity both in the short term and the long term.

The idea that single-sport training at young ages increases the prospect of future sport success has been popularized in the media, but there are few scientific data to support this approach. Yet, there is a fear among parents, coaches, and young athletes that not specializing in one sport early will place the child at a competitive disadvantage. In fact, research indicates that early sport specialization is not a pre-requisite and may even be detrimental to long-term achievement and elite performance [5–14]. There is also a concern that excessive focus on sport-specific intensive training and competition at a young age may impede an athlete’s ability to develop transferable athletic skills, and possibly increase the risk of burnout and overuse injury, rather than optimize participation and foster interest in a variety of sports [15–20]. Regarding the relationship between injury and early single-sport specialization, the data at this time are limited and do not provide consistent evidence [21–26].

## Aim and Procedure

In 2016, the Jr. NBA partnered with USA Basketball to address issues facing youth basketball in the USA. As part of this initiative, a multidisciplinary team of clinicians and researchers with expertise in athlete development and youth sports was assembled.

This group assessed the existing research related to youth sport participation, focusing on the sport of basketball. A series of seven meetings were held from May 2016 to July 2017 to review these data. From this, the recommendations for best practice in youth basketball were developed. Each recommendation was classified using the Strength of Recommendation Taxonomy system (SORT, Table 1) [27].

**Table 1:** Strength of recommendation taxonomy (SORT)

Strength of recommendation	Basis for recommendation
A	Consistent, good-quality, patient-oriented evidence
B	Inconsistent or limited-quality patient-oriented evidence
C	Consensus, disease-oriented evidence, usual practice, expert opinion, or case series for studies of diagnosis, treatment, prevention, or screening

## Basketball Participation

Basketball has high levels of participation for girls and boys across all age ranges, including recreational play and organized competition. Among US youth 6–14 years of age, 14.4 million play basketball, representing 39% of this age group [28]. Furthermore, basketball is the most popular team sport for those 12–17 years of age, with over 11 million participants. At the high school level, approximately 430,000 girls and 550,000 boys play interscholastic basketball [29]. Importantly, the top reason for playing basketball, cited by 74% of children and adolescents, is to have fun [30]. Basketball is a sport that can be modified so that it can be played informally in groupings of one, two, or three players on a side (i.e., one-on-one, two-on-two, or three-on-three). In fact, 50% of children and adolescents cite that one of the reasons they started to play basketball was because it can be played with any number of people [30]. Such recreational play is also a reason that the game can be enjoyed into adulthood. In addition, wheelchair basketball is a team sport for individuals with chronic conditions resulting in lower-limb disability such as spinal cord injury, cerebral palsy, musculoskeletal conditions, spina bifida, amputation, and poliomyelitis, and a reduced ability to play running basketball in the same manner as able-bodied players [31, 32].

## Basketball Promotes Healthy Youth

In addition to the psychosocial benefits described above, youth sports can provide participants with other health benefits, including those involving the cardiorespiratory, musculoskeletal, and metabolic systems [33–39]. While physical activity is essential for healthy childhood growth and development, children in the USA and globally are not sufficiently active [40]. Recent research has shown that the development of fundamental movement skills (FMS) in children is linked to lower levels of overweight, and higher levels of physical activity, cardiorespiratory fitness, and self-esteem [41]. Mastery of FMS such as the sprint run, vertical jump, and overarm throw, has been shown to be low [41, 42]. However, the implementation of FMS programs in schools is effective in improving FMS competencies [42].

Basketball promotes speed, agility, strength, power, endurance, flexibility, and motor coordination. As a result, basketball is uniquely oriented to improve FMS, and has been shown to be beneficial in promoting general health. In one study, basketball, along with soccer and track, provided middle school children the highest level of physical activity, regardless of the way schools offered the sport [43]. This is important in light of public health concerns related to obesity and diabetes among youth, while paradoxically, participation in school-sponsored physical education programs is low [44]. Specifically, the study suggested that basketball can effectively increase physical activity and reduce the long-term negative health consequences of an inactive lifestyle, while being an efficient option in the face of limited school resources.

Basketball can also have a positive effect on bone mineral density (BMD) for boys and girls [45–47]. A prospective study of teenage girls compared basketball players to age-matched controls and found that those who played basketball had significant increases in BMD [48]. This is important since maximizing BMD at these ages provides the basis for long-term bone health throughout adulthood [49].

There is also evidence that health benefits obtained via youth sports activity can extend into adulthood [50–54]. For example, physical activity during adolescence predicts lower cardiometabolic risk in adulthood [55]. In addition, youth sport participation appears to be associated with better mental health in later life [56]. Importantly, because basketball can be modified to allow participation in various small-sided formats, it is a sport that is conducive for participation well into adulthood, thus yielding health benefits over a wide age range.

## Injuries in Youth Sports: How Does Basketball Compare?

Among youth sports, basketball has a relatively low injury rate. A decade-long surveillance study of US high school sports found that basketball consistently had lower injury rates than football, wrestling, and boys’ and girls’ soccer [57]. With respect to overuse injuries, basketball has a relatively low injury rate at the high school level [58]. In fact, in a study of high school sports, basketball had the lowest overuse injury rate in boys and the second lowest rate in girls [58]. In addition, among female middle school athletes, basketball had a lower injury rate than both soccer and volleyball [59].

## Injury Risk Factors and Injury Prevention in Youth Basketball

### Risk Factors

Several risk factors for injury in youth sport have been identified, though data specific to basketball are limited. Prior injury, low energy availability, and training volume have been shown to be important risk factors. Previous sport-related injury is perhaps the most-established predictor of subsequent injury [60–62]. Low energy availability, a relative deficit in energy needs, may increase the risk of bone stress injuries in both boys and girls [63, 64]. Bone stress injuries that are a result of low energy availability highlight the dangers of excessive training and competition, especially when combined with inadequate provision for re-fueling and recovery [63–65]. A weekly training time of > 16 h per week among 14- to 18-year-old youth has been correlated with injury risk [66–68]. As in most sports, the injury rate in basketball is greater in competition than practice [69, 70]. In addition, youth athletes who participated in organized sports compared to peer-led play at greater than a 2:1 ratio were found to have an increased injury risk [22, 71]. However, the actual risk associated with different amounts of participation still needs validation [22, 71]. In addition to training volume, the risk of injury may be greater during the adolescent growth spurt, though further study is needed [15, 16].

It is not clear if these data are generalizable to basketball or to more structured sport training settings. Research is also needed to guide long-term, sport-specific development programs.

### Injury Prevention

Data on injury prevention programs for sports in general and for basketball in particular are limited. In addition, very little research has focused specifically on injury prevention among young athletes. Aimed at providing youth athletes with a standardized warm-up designed to prevent non-contact knee and lower extremity injuries in soccer, the original FIFA 11 program and the more recent FIFA 11+ modification have had a favorable effect in decreasing certain soccer injuries [72–75]. The program consists of 15 exercises that include running, active stretching, core strength, balance, and agility. A recent study using the FIFA 11+ program in high-level European basketball players also reported a reduction of injury in several categories [76]. A similar neuromuscular training program has been shown to be effective in high school basketball players [77].

Other studies have focused on improving balance to decrease injury rates. Such studies have included adolescent and professional basketball players, and have been shown to be effective in reducing acute injuries including ankle and knee sprains, as well as back injuries [78, 79]. A program aimed at preventing hamstring injuries has been validated in soccer, but has not yet been studied in basketball [80].

Strength and conditioning programs may play a role in injury prevention as well. These programs can be safely performed by young athletes if properly implemented and supervised. In particular, preseason conditioning programs appear effective in reducing injuries [81–87].

An often-overlooked component of athlete development and injury prevention is rest. In a study of high school athletes, a 42% increase in self-reported overuse injuries was noted among those who participated all year compared to those who trained in three or fewer seasons per year [88]. At least one rest day per week, and additional periods of time away from organized sports, are recommended for physical recovery and to avoid burnout [15, 16]. In addition, sports events or “tournaments” that involve more than one full-length competition per day, in some cases for multiple consecutive days, may in some circumstances increase injury risk further due to the high-volume loading coupled with limited recovery time [15, 16].

Thus, neuromuscular training programs, including a modified FIFA 11+ program, appear promising for reducing lower extremity injuries and should be considered for broader implementation trials in youth basketball. Recent consensus statements from the American Orthopedic Society for Sports Medicine (AOSSM) and the International Olympic Committee (IOC) are supportive of such measures [71, 89]. Measures that focus on monitoring and managing training volume—including scheduled rest and recovery, ensuring proper treatment when injuries occur, and addressing issues of relatively low energy availability and bone health—are warranted [64]. Injury prevention programs aimed at reducing hamstring injuries appear valid but need further study in basketball players.

## Early Single Sport Specialization—The Road to Success?

Single sport specialization can be defined as intensive year-round training in one sport to the exclusion of others [90]. A perception exists among parents, athletes, and coaches that early single-sport specialization is necessary for long-term success. This can lead to a focus on short-term results at young ages rather than the overall development process.

The concept of early sport specialization was popularized in the USA more than 20 years ago based upon studies of chess players and musicians, but not athletes [91]. The central tenet of this model is that an individual’s ultimate level of performance is directly related to the accumulated amount of deliberate practice (DP). The authors advocated the maximization of DP, which implies an early start, intensification, and subsequent expansion of DP. It was suggested that 10 years of DP is needed to achieve the highest performance levels [91].

In contrast, the state of empirical research in athletes does not provide much support for these perceptions. Several studies have shown that competitive success at the youth level correlates modestly at best, or not at all, with long-term senior success [8, 9, 92–94]. That is, early success has been described as neither a necessary precondition nor a valid predictor of long-term success.

A recent review highlighted that the participation patterns that likely lead to youth success are not the same as those that facilitate long-term development and adult success [95]. Short-term youth success is indeed correlated with early single sport specialization and intensified, sport-specific practice/training during childhood (age ≤ 12 years) and adolescence (13–18 years) [9, 96–102]. In contrast, *adult* world-class athletes from all Olympic sports and different countries typically engaged in only *moderate levels of early* practice/training intensity in their respective primary sport. Reports of world-class athletes in basketball, field hockey, and soccer show that they attained international success accumulating much less than 10,000 practice h, specifically 4000–4500 h [11, 12, 81]. In this context, world-class athletes (e.g., Olympic and World Champions, medalists or top-ten athletes) did not differ from national-class peers in terms of the amount of sport-specific youth practice/training [5, 9, 11, 12, 95, 103–105]. Interestingly, several studies indicate that eventual world class athletes had a relatively lower level of sport-specific training during childhood [5, 9, 103, 106]. Further, international-level performers typically participated in a diverse set of sport activities, including peer-led play, and organized practice in various sports. Importantly, world-class athletes were more likely than national-class peers to engage in multiple sports [13, 95, 106]. These athletes *specialized* in their primary sport significantly *later* than their national-class peers [5, 9–12, 106]. These findings have been confirmed even when comparing Olympic and World Championship medalists to non-medalists [13, 106]. These findings were also consistent across different countries and types of sports, and were confirmed in a 3-year prospective study [9].

Further, closer scrutiny of the “micro-structure” of practice of German world-class soccer players highlighted the significance of *play*. Within their total childhood soccer activities, only 14% involved drill-like training of technical skills or physical conditioning. As much as 86% was a combination of coach-led play (17%; including conditioned, small-sided games), and peer-led play (69%; “kicking around with friends”) [11].

These observations do not diminish the critical significance of organized sport-specific practice on specific skill development. However, it should be recognized and placed into perspective that early reinforced intensification and specialization is unnecessary and may even be detrimental to long-term success. Alternatively, the interaction of sport-specific practice with multisport practice and play facilitates long-term development [9, 17, 19, 90, 106, 107].

The preceding discussion presumably relies on the interplay of three processes. First, in addition to training volume *per se*, single-sport specialization may constitute an independent risk factor of overuse injury. Diversified involvement may reduce susceptibility to overuse injury, presumably due to less cumulative stereotypical mechanical impact on certain tissues and may promote prolonged participation [15, 17, 19, 22, 24, 90, 107]. Second, youth who have explored various sports may make the decision to invest in one primary sport based on their own experiences in different sports. This likely enhances the probability that a child or adolescent elects a primary sport that optimally “fits” him or her (where “optimal fit” may represent talent at a particular sport, experienced performance progress, enjoyment, health, social interaction, etc.) [8, 9]. Third, physical conditioning and perceptual-motor and psychological skills can be directly transferred across related sports [108–112]. Perhaps more importantly, early *variable learning experiences* improve the *efficacy* of (later) practice within the primary sport (greater performance improvement per invested practice time) [5, 9, 11, 12, 14, 106, 113]. Athletes acquire a multifaceted repertoire in terms of a wider and closer-meshed “network” of perceptual-motor skills, which facilitates the emergence of functional skill solutions [95, 114]. *Play*, in particular, unlike drill-like practice exercises, involves the interaction of situation dynamics, perception, and motor solutions, and also provides extensive implicit skill learning. This may lead to more robust skills exhibiting less susceptibility to physiological or psychological stress and better retention [115, 116]. Peer-led play, for its part, may further amplify tasks and situations (playing different roles/positions, varying rules, surfaces, court sizes, balls, number and skill level of participants) [95]. Moreover, exploring varying *practice designs* and *learning modes* can facilitate the development of individual functional *learning solutions*, leading to more adaptive, “smarter learners” [95, 106, 113]. In this context, it is important to note that the multisport participation of world-class athletes constituted *authentic* experiences in that it typically included multi-year *competition*-related engagement—i.e. long-term dedicated, performance-related learning processes with specialist coaches in broadened ranges of tasks and situations [9, 11, 12, 14, 106].

Consistent with this discussion, a number of reviews and position statements have highlighted the potentially negative effects of early specialization and the positive impact of diversified youth experiences among sports and settings [13, 15, 16, 71, 83, 90, 117–124]. This reinforces the idea that childhood/adolescence multisport engagement facilitates long-term performance development—in association with positive health and psychosocial development. At the *program* and sport *system* level, this contributes to the growth of prolonged youth sport participation and expands the potential pool of talented youth athletes. In contrast, reinforcing early specialization likely diminishes general participation and the “talent pool.”

## Personal Engagement as a Model for a Positive and Successful Youth Basketball Experience

The findings described above have also been highlighted in applied frameworks informing policy-makers and stakeholders of the sport system, such as FTEM (Foundations, Talent, Elite, Mastery) or the DMSP (Developmental Model of Sport Participation) [6, 118, 125, 126]. The practitioner-derived FTEM highlights the socio-environmental, organizational, and sport-system requirements and applications, while the DMSP more particularly looks into the psychosocial influences and outcomes in terms of positive youth development. Both frameworks emphasize the foundational role of early diversified involvement for either developing sporting excellence or prolonged recreational engagement. This section focuses on the DMSP.

The DMSP posits that personal engagement in sport grows from involvement in sport activities, relationships, and environments that evolve throughout development [127]. Combining personal engagement in sport with early sport sampling promotes a rewarding youth experience and long-term sport success [128]. For either recreational or competitive basketball, personal engagement is a primary objective of participation during youth. For this to occur, resources to develop personal meaning are needed, including: access to appropriate sport environments and role models; activities that provide personal relevance; a positive social climate; encouragement in the face of difficulty; opportunities for leadership, challenge, and knowledge-building; and opportunities to feel in-control, competent, and connected with others [129–134].

Within the DMSP, the sampling years lay an important foundation for youth to achieve optimal outcomes in sport over time [130]. Sampling generally begins during childhood, and is characterized by participation in a variety of different sports, as well as different activities within a given sport (e.g., peer-led play, organized coach-led practice). Following the sampling years, athletes may continue to participate in sport at a recreational level or begin to invest more and perhaps specialize in one sport during adolescence or later.

At any stage within the DMSP, youth may choose to disengage from sport; however, nurturing individual capacities for personal engagement throughout development enhances opportunities for physical and psychosocial development. By focusing on the personal, social, and physical features of different activities (e.g., interest, play, practice, sampling, specialization) across development, the DMSP suggests that the positive outcomes of sport result from the integration of processes that include personal engagement in a sport activity, the social relationships that are formed within this activity, and the physical environment in which this activity takes place [135]. More recently, the features of the DMSP have been integrated with previous youth sport research and principles from developmental systems theories to create the Personal Assets Framework for sport (PAF) [136–138].

The PAF is, in essence, a set of key elements that should be combined to provide quality sport programs for youth that not only contribute in a positive way to the overall development and well-being of the person, but also to the development of talent in sport. In line with developmental systems theories, the PAF considers personal (i.e., personal engagement in activities), relational (i.e., quality relationships), and environmental factors (i.e., appropriate social and physical settings) as the elements necessary to understand the mechanisms through which development occurs in and through sport. The interaction of the three dynamic elements constitutes a specific sport experience—for example, a game, practice, or team social activity. When repeated over a period of time, such as the span of one season, the specific sport experiences generate changes in an athlete’s personal assets (e.g., confidence, competence, connection, and character) and provide personal meaning to the sport being practiced. Eventually, changes in the personal assets will influence the long-term outcomes of sport in relation to the individual’s participation, performance, and personal development [139, 140].

The PAF highlights the dynamic elements and personal assets that should be combined in youth basketball programs that promote performance, participation, and personal development. Different lines of research on sport expertise and youth sport demonstrate that the objective of elite performance and continued participation are not mutually exclusive during childhood and that effectively designed sport programs for children can contribute to the overall development of youth in sport [141].

Two concepts regarding sport involvement throughout the lifespan consistently emerged from the empirical data that support an early sampling approach: diversity and peer-led play [136]. Firstly, the concept of diversity describes a level of involvement in different types of sport experiences during childhood (e.g., participating in different sport activities, or playing different positions within a sport activity), before specializing and intense training in one sport. Secondly, the concept of peer-led play relates to the notion that elite-level athletes engaged in sport activities during childhood that were inherently enjoyable and differed from organized sport and adult-led practices [136]. Peer-led sport-play activities represent a distinctive form of sport activities that add to the breadth of contexts and experiences of the youth sport environment. Together, the concepts of diversity and peer-led play form the backbone of the sampling years and may have a protective effect against burnout, dropout, and/or injuries [17–19, 142].

At a population level, youth sport programs that focus on diversity before specialization and play before practice may better maximize the potential impact that youth sport activities can have on youth development and long-term performance in sport. As suggested by the different pathways of the DMSP, the diversity and play aspect of sport activities during the sampling years should not be viewed as a discriminating factor that predicts sport expertise, but rather as a foundation for optimal development in an elite performance or recreational pathway. The nurturing of talent through sampling without an intense focus on performance in one sport during childhood can have more positive outcomes and less negative consequences for all children involved in sport, while still facilitating the development of expertise.

## Growth, Maturation, and Readiness for Basketball

Sport readiness is the relationship between a child’s stage of growth and development and the physical and cognitive requirements of that sport [143]. Understanding that motor skills as well as social and emotional development influence a young athlete’s ability to perform physical tasks and to understand instructions is essential to promote a rewarding experience. Given inter-individual variability, chronologic age is not a reliable marker for these development levels [15].

It is clear that if a child is expected to learn too many skills that are beyond his or her ability, the child may become less motivated to learn new skills, and may eventually cease participation in the sport [144]. Conversely, a child who begins to master new tasks will develop a feeling of competence that may motivate further skill acquisition, and further interest in the sport [143].

Coaches and parents who are not aware of these issues may unintentionally create unrealistic expectations that can cause children and adolescents to feel as if they are not making progress, especially compared to chronological peers who may simply be at a different stage of growth and maturation. This, in turn, can result in loss of self-esteem and sport discontinuation [144].

Although there is no straightforward way to determine if a child is ready for basketball or another sport, important factors to consider include sport-related skills, knowledge about the sport, motivation, and socialization [142, 145]. Parents and youth coaches should recognize the need to nurture young athletes. It is important to appreciate that even for talented individuals, the ups and downs along the path might be more related to biological maturation than to specific coaching and training techniques [123].

## Conclusions

Basketball, both competitive and recreational, is a sport that has many positive attributes with respect to health and wellness. It involves moderate to high levels of sustained activity, has a relatively low injury rate, engenders positive psychosocial interactions, and is perceived as a fun game to play. The last point is significant in that it encourages long-term involvement, which in turn provides for benefits that extend into adulthood.

### Recommendations

Based upon the preceding review of the literature and the consensus of this working group, the NBA and USA Basketball offer the recommendations described in Table 2 for young athletes, parents, coaches, and basketball organizations. Each recommendation is graded using the SORT system [27].

**Table 2:** Recommendations for youth basketball participation

Topic	Recommendation	Strength^a^
Personal engagement	*Promotion of personal engagement should be a priority in youth basketball.* Sport programs that invest in providing opportunities for youth to connect with others, build relationships, and take on challenges and leadership roles promote overall personal development, well-being, and talent development	B
Multisport engagement and delayed specialization	*Youth should be provided opportunities and be encouraged to engage in diverse sports and delay single-sport specialization.* Multisport engagement through childhood and adolescence is associated with reduced risks of overuse injury, and facilitates prolonged participation, psychosocial development, as well as long-term talent development. World-class athletes in basketball and other sports often delayed single-sport specialization until age 16 years or later. It is recommended that specialization for basketball be delayed until this age. However, given that the age of high-school entry in the USA is typically age 14 years, the working group recognizes that specialization may occur at this time. However, specialization prior to age 14 years is discouraged	B
Varied settings	*Youth should be provided opportunities and be encouraged to engage in both organized, coach-led basketball activities and peer-led play.* Peer-led basketball play allows youngsters to experience largely self-determined, intrinsically motivated activity, be creative and challenge themselves. It may contribute to individual growth and long-term talent development	B
Rest and time off	*Coaches and parents should ensure sufficient rest and time away from organized basketball practices and competitions.* It is recommended to ensure a minimum of one day of rest each week and multiple months per year away from organized basketball. Proper daily sleep, rest days, and off-periods provide physical recovery, reduce injury risks, and further psychological recharging	C
Competition density and cumulation	*Cumulative, high-density competitions should be avoided.* High-density competition scheduling may increase injury risk and fatigue, and lead to loss of motivation. Parents, coaches, event directors and administrators should be cautious in designing basketball events. The working group recommends a maximum of two games per week per player through childhood, and a maximum of three games per week through late adolescence. The working group also recommends reducing game duration for tournaments or events during which multiple high-density competitions are scheduled (i.e., reduce the number of minutes per game). “Rest games,” where some players are rested while their teammates compete, together with larger roster sizes, will allow teams to participate without overloading individual players	C
Injury prevention programs	*Neuromuscular injury prevention programs should be implemented and evaluated.* Such programs have been shown to reduce lower extremity injury. However, further evaluation of basketball-specific programs is warranted	B
Sport readiness	*Parents and coaches should adjust demands to the individual player’s development.* Individuals develop at different rates. Moreover, through adolescence in particular, physical, motor, cognitive, emotional, and social development may proceed asynchronously within one player. Carefully adjusting expectations and demands to the individual player’s development furthers a rewarding experience, progress in learning, motivation, and a healthy life balance	B

^a^Each recommendation in this table has been classified using the Strength of Recommendation Taxonomy system (SORT) defined in Table 1 [27]. Recommendations of strength B are based on inconsistent or limited-quality patient-oriented evidence and recommendations of strength C are based on expert opinion consensus

The following guidelines (Tables 3, 4, 5) are based upon the consensus recommendations of the NBA and USA Basketball working groups on Playing Standards and Health & Wellness. These guidelines draw on the available scientific evidence at this time, as well as the expert opinion of the working groups and current and former men’s and women’s players, coaches, and administrators from all levels of basketball. These recommendations may need to be updated as new research and information develops.

**Table 3:** Recommended participation guidelines

Age (years) or grade	Game length (min)	No. of games per week	Practice length (min)	No. of practices per week
Ages 7–8	20–28	1	30–60	1
Ages 9–11	24–32	1–2	45–75	2
Ages 12–14	28–32	2	60–90	2–4
Grades 9–12	32–40	2–3	90–120	3–4

**Table 4:** Maximum participation guidelines

Age (years) or grade	No. of games per day	No. of hours per week in organized basketball^a^
Ages 7–8	1	3
Ages 9–11	2^b^	5
Ages 12–14	2^b^	10^c^
Grades 9–12	2^b^	14^c^

^a^Organized basketball includes game competition and practice time and structured training in which an athlete works in a focused way to improve his or her game, typically with or at the direction of a coach. Unstructured peer-led on-court activities do not constitute organized basketball for the purpose of this table (e.g., pickup games, a player shooting baskets by themselves, a player working with a peer to practice a skill). Youth basketball *camps* can be a positive experience for young players. Camp program content and duration is variable and may exceed the practice guidelines above. Camp directors should, however, keep the above guidelines in mind, and seek to include activities other than on-court basketball as well as rest days. The research team also recommend additional rest days following camp attendance. Residential youth sport *academies* also exist, particularly outside the USA. Studies in Europe point to earlier specialization, enhanced specific practice intensity and increased risks of impaired well-being, health and academic performance in the sport-students [147]. Therefore, attention to these issues is warranted. As such, academy directors and coaches should recognize the risks of early specialization and benefits of diversified participation. Their sport curricula should involve activities other than basketball to a significant portion up to age 14 years or beyond, including both organized and non-organized settings

^b^ Youth basketball players, parents, and coaches should demonstrate caution in scheduling or participating in more than one game per day, especially on consecutive days. If young athletes participate in an event or tournament in which more than one game is played per day on consecutive days, players should have additional time off from sports activities following the event to allow for recovery

^c^It is recommended that young athletes in these age ranges who are approaching the maximum recommended hour limits do not participate in another sport concurrently

**Table 5:** Rest guidelines

Age (years) or grade	Minimum no. of rest days per week	Maximum months per year in organized basketball	Recommended hours of sleep per night [148]
Ages 7–8	2	4	9–12
Ages 9–11	2	5	9–12
Ages 12–14	1	7	8–10^a^
Grades 9–12	1	9–10	8–10

^a^For 12-year-olds, 9–12 h of sleep is recommended

### Implementation and Future Directions

The NBA and USA Basketball are committed to driving positive change in youth basketball that promotes a healthy and positive experience for players. Efforts aimed at the grass roots level is essential for this to occur. To achieve this, these guidelines are now being implemented across their youth programming, and they have partnered with key organizations across youth basketball to similarly endorse and adopt the guidelines. Further, the NBA launched in October 2017 the Jr. NBA Flagship Network to provide a more consistent and positive youth basketball experience for players, parents, and coaches. Members of the network include 15 best-in-class organizations that share the Jr. NBA’s vision for how the game should be taught and played at the grassroots level [146]. They have committed to adhering to NBA and USA Basketball Youth Guidelines, including USA Basketball coach licensing requirements and providing resources to educate coaches and parents. Finally, the NBA and USA Basketball have begun to assess the extent of basketball participation among youth, the adoption of the basketball guidelines, and the response to this initiative in youth basketball.
